# Structural basis of substrate specificity in human cytidine deaminase family APOBEC3s

**DOI:** 10.1016/j.jbc.2021.100909

**Published:** 2021-06-24

**Authors:** Shurong Hou, Jeong Min Lee, Wazo Myint, Hiroshi Matsuo, Nese Kurt Yilmaz, Celia A. Schiffer

**Affiliations:** 1 Department of Biochemistry and Molecular Pharmacology, University of Massachusetts Medical School, Worcester, Massachusetts, USA; 2 Basic Research Laboratory, Frederick National Laboratory for Cancer Research, Frederick, Maryland, USA

**Keywords:** APOBEC3, specificity, structural analysis, molecular modeling, molecular dynamics simulation, CBE, cytosine base editor, CTD, C-terminal domain, NTD, N-terminal domain, pMD, parallel molecular dynamics, ssDNA, single-strand DNA

## Abstract

The human cytidine deaminase family of APOBEC3s (A3s) plays critical roles in both innate immunity and the development of cancers. A3s comprise seven functionally overlapping but distinct members that can be exploited as nucleotide base editors for treating genetic diseases. Although overall structurally similar, A3s have vastly varying deamination activity and substrate preferences. Recent crystal structures of ssDNA-bound A3s together with experimental studies have provided some insights into distinct substrate specificities among the family members. However, the molecular interactions responsible for their distinct biological functions and how structure regulates substrate specificity are not clear. In this study, we identified the structural basis of substrate specificities in three catalytically active A3 domains whose crystal structures have been previously characterized: A3A, A3B- CTD, and A3G-CTD. Through molecular modeling and dynamic simulations, we found an interdependency between ssDNA substrate binding conformation and nucleotide sequence specificity. In addition to the U-shaped conformation seen in the crystal structure with the CTC_0_ motif, A3A can accommodate the CCC_0_ motif when ssDNA is in a more linear (L) conformation. A3B can also bind both U- and L-shaped ssDNA, unlike A3G, which can stably recognize only linear ssDNA. These varied conformations are stabilized by sequence-specific interactions with active site loops 1 and 7, which are highly variable among A3s. Our results explain the molecular basis of previously observed substrate specificities in A3s and have implications for designing A3-specific inhibitors for cancer therapy as well as engineering base-editing systems for gene therapy.

APOBEC3s (A3s) are a family of cytidine deaminases that have seven members in human with functions in innate immunity and roles in cancer (1, 2, 3, 4, 5). All A3 domains share a conserved structural fold with an active site zinc tetrahedrally coordinated with catalytic His and Cys residues and an additional water. The human A3s have either one (A3A, A3C, and A3H) or two zinc-binding domains (A3B, A3D, A3F, and A3G). The two-domain A3s consist of a catalytically active C-terminal domain (CTD) and an N-terminal domain (NTD) that binds to substrate but has no deamination activity. A3s deaminate cytosine to uracil on single-strand DNA (ssDNA) and certain RNAs (6, 7), thus creating mutations.

Through deamination, A3s play crucial roles in innate immunity by mutating foreign pathogenic genomes and thus protecting host cells against retroviruses and retrotransposons (8, 9, 10, 11, 12, 13, 14). Specifically, A3s deaminate cytosines to uracils on ssDNA during reverse transcription to create G to A hypermutations on the complementary strand. However, misregulated A3 deamination activity may promote cancer and the development of therapeutic resistance. Overexpressed A3s, especially A3A, A3B, and A3H, have been shown to cause heterogeneities in multiple cancers, including breast, bladder, head and neck, cervical, and lung cancer (15, 16, 17, 18). The A3 mutational signature, which is C to T transition in TC context, has been observed in multiple cancer genomes (15, 16, 19). Moreover, study of human cancer cell lines has suggested that A3s may be involved in the origination of cancer (20). Recently, coupled with CRIPSR/Cas9, A3s are explored as novel base editors to treat genetic diseases (21, 22).

The structures of A3s provide the basis for understanding the underlying molecular mechanisms in A3 biology. Several crystal and NMR structures of human or primate A3 single domains (A3A, A3C, A3H; CTDs of A3B, A3F, A3G; NTDs of A3B, A3G) in the apo state have been determined by our group (23, 24, 25, 26, 27, 28, 29) and others (30, 31, 32, 33, 34, 35, 36, 37, 38, 39, 40, 41, 42, 43, 44, 45, 46). The A3 domain fold consists of six alpha-helices and five beta-strands. The catalytic active site, which also coordinates zinc binding, is in the middle of the domain (Fig. 1*A*). Recently, our laboratory (47, 48), along with other groups (48, 49), has solved the crystal structures of several A3–ssDNA complexes (A3A-DNA, chimeric A3B-CTD–DNA, A3G-CTD–DNA). These structures identified the binding conformation of DNA, revealed the critical residues for binding, and provided insights into substrate specificity, especially at the −1′ position upstream of the target C. Of these structures, three (A3A, A3B, and A3G) have substrate DNA bound at the active site, with the target cytidine to be deaminated in essentially the same conformation. However, the rest of ssDNA can bind to A3 in different conformations; either in a conformation wrapped around the gatekeeper residue, U-shape, as seen around His29 in A3A (PDB: 5KEG; 5SWW) (47, 49) and chimeric A3B-CTD (PDB: 5TD5) (49), or a more extended linear shape, L-shape, as seen in A3G-CTD (PDB: 6BUX) (48), (Fig. 1). As these complex structures were determined with varying ssDNA sequences, the conformation of the bound DNA may determine the differential specificity of these enzymes.

**Figure 1:**
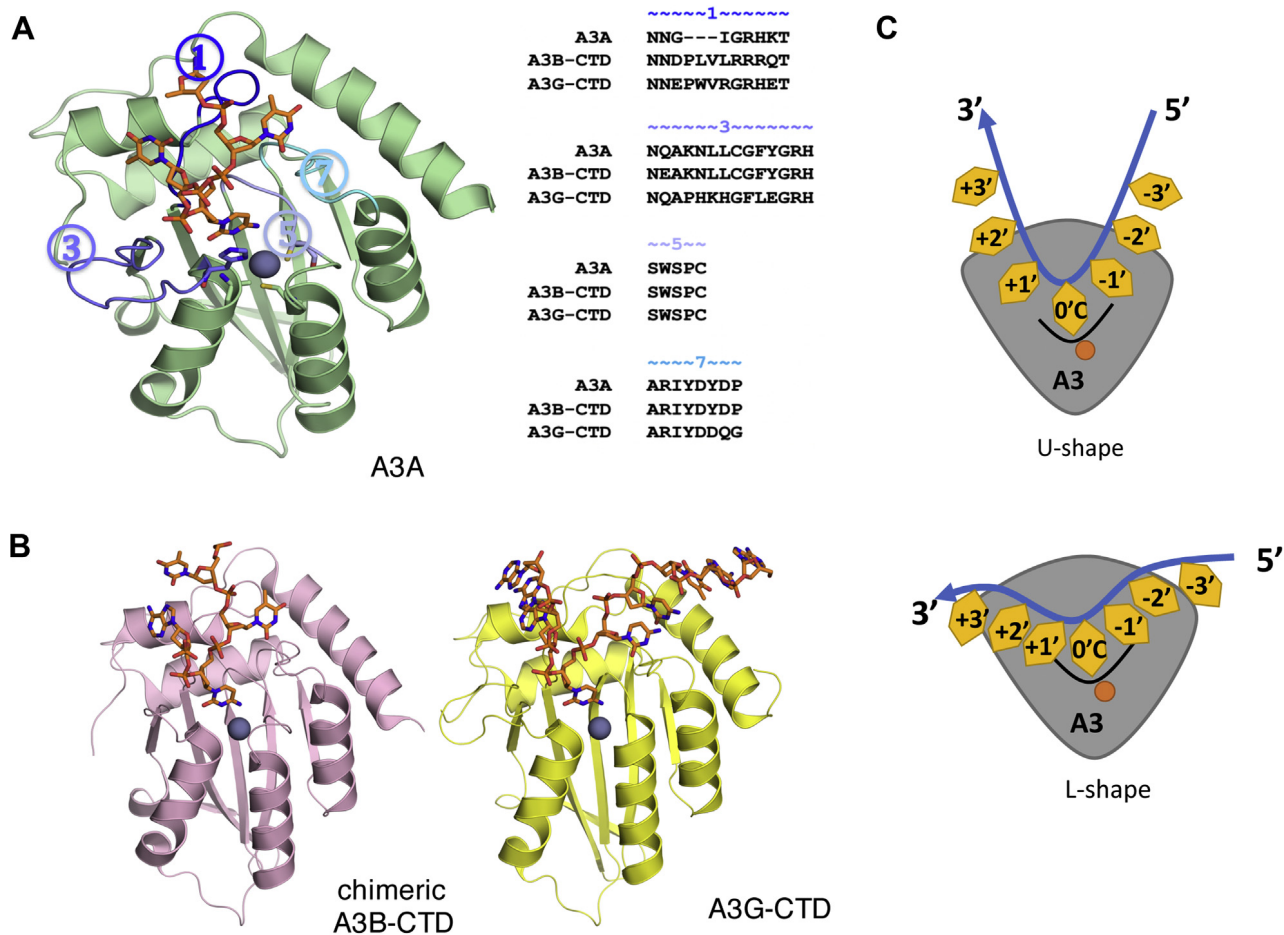
**Structure and active site loops of A3s.***A*, crystal structure of ssDNA-bound A3A where the active site loops 1, 3, 5, and 7 are highlighted (PDB ID: 5KEG). ssDNA (*orange*) and the residues coordinating the zinc (H70, E72A, C101, and C106) are shown as *sticks*. The catalytic zinc is shown as *gray sphere*. Amino acid sequence alignments of active site loops in A3A, A3B-CTD, and A3G-CTD are given on the *right*. *B*, crystal structure of chimeric A3B-CTD (PDB ID: 5TD5) and A3G-CTD (PDB ID: 6BUX) in *cartoon* representation. *C*, schematic representation of the ssDNA binding conformations: U-shape as seen in A3A or chimeric A3B-CTD, or a more extended linear (L) shape as seen in A3G-CTD. The target C is in the zero (0) position with the active site zinc indicated by the *dark orange circle*, and the other nucleotide positions are represented by orange diamonds along the ssDNA from 5′ to 3′ direction (*blue arrow*).

Although A3s share highly similar overall structural folds, they have varying levels of deamination activity and substrate specificity. For instance, the activity of A3A, which is the highest in A3 family, can be up to 5000-fold higher compared with the least active A3D (50). All A3 proteins deaminate deoxy-cytidines in ssDNA, but vary in their preferred hotspot sequences or deamination motifs: 5’- (T/C)TC(A/G) for A3A, 5′-ATC(A/G) for A3B and 5′-CCC(A/C/T) for A3G (50, 51, 52, 53, 54). However, based on the amino acid sequence or even the available structures, the molecular mechanisms that are responsible for varied ssDNA binding affinity and deamination activity as well as substrate specificities among A3 domains are not apparent. The loops (loop 1, 3, 5, and 7) surrounding the active site pocket are the least conserved region of A3s (Fig. 1*A*; Fig. S1). In addition, these active site loops undergo substantial conformational changes relative to apo structures upon ssDNA binding. Therefore, changes surrounding the active site including variations in the arrangements of these loops are likely largely responsible for distinctions in substrate specificity, binding affinity, and deamination activity for ssDNA, as well as the physiological functions of A3s. Structural analysis using molecular modeling and dynamics simulations have been used for investigating the molecular mechanisms underlying biological functions of A3s (55, 56, 57, 58, 59, 60). These studies have revealed the importance of active site loops in substrate binding/activity and specificity. However, the structural mechanism for substrate specificities in A3s is largely unknown.

In this study, we investigate the structural mechanism of substrate specificity and ssDNA-binding conformation in A3s using a combination of molecular modeling, structural analysis, and parallel molecular dynamics (pMD) simulations (7, 61, 62, 63, 64, 65). Three domains of the human A3 family that have high enzyme sequence similarity yet varied substrate specificity are the focus of this study: A3A, A3B-CTD, and A3G-CTD. These enzymes are the best experimentally characterized A3s in terms of available structures and substrate specificity (Figs. 1 and 2) (47, 48, 49, 61). In our analyses, A3 domains were characterized bound to ssDNA of varying nucleotide sequences in either U or L-shape conformation, and the results show an interdependence between substrate specificity and ssDNA conformation. Although the ssDNA was crystallized in a U-shape with A3A and (chimeric) A3B-CTD (47, 49), we find that the A3A-ssDNA enzyme complex adopts varied conformations in a substrate dependent manner, binding ssDNA with a CTC_0_ motif in a U-shape and CCC_0_ in an L-shape, A3B can accommodate either conformation in the recognition of its preferred ATC_0_ substrate motif, while A3G only stably recognizes CCC_0_ with the ssDNA in an L-shape. These sequence-specific conformations are stabilized by specific interactions with the highly variable loops, loop-1 and loop-7, of the A3 enzymes (Fig. 1). Thus, A3 family of enzymes accommodate varied substrate specificity through conformational rearrangements of both the active site loops and the ssDNA substrate.

**Figure 2:**
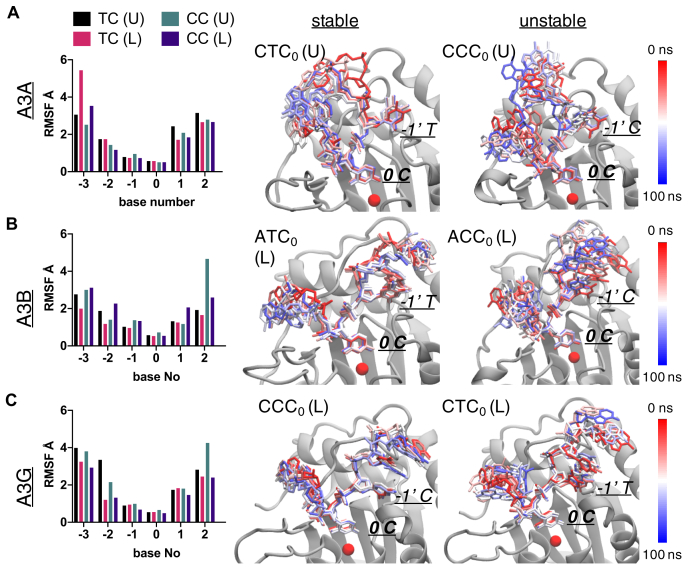
**Differential dynamics of ssDNA during the pMD simulations elucidate the stable substrate-bound A3 complexes.** The root-mean-squared fluctuations (RMSF) of each nucleotide in four complexes with different sequences (TC or CC) and binding conformations (U or L) are shown as histograms. Two examples of stable (*middle column*) and unstable (*right column*) complexes for each of (*A*) A3A, (*B*) A3B-CTD, (*C*) A3G-CTD are depicted. In each panel, seven snapshots of the ssDNA conformation evenly spaced throughout a 100 ns MD trajectory are shown. The A3 proteins are in *gray cartoon* representation. The ssDNA is shown as *stick*; colored based on the simulation time from *red* to *blue*. The target (0 C) and −1′ position nucleotide in the active site are labeled. The catalytic zinc is shown as *red sphere*.

## Results

The three A3s investigated, A3A, A3B-CTD, and A3G-CTD, were modeled and energy minimized with the DNA conformation either in a wrapped (U) or linear (L) shape based on that in the cocrystal structures with A3A and A3G-CTD (47, 48) respectively (Table S1). The preferred trinucleotide deamination motif is (C/T)TC_0_ for A3A, ATC_0_ for A3B-CTD, and CCC_0_ for A3G-CTD (50, 51, 52, 53, 54). For each A3 enzyme the preferred motif of substrate DNA in both the U and L conformation were characterized. The complexes investigated together with full ssDNA nucleotide sequences are tabulated in Table S1. To analyze the relative stability of these A3–ssDNA complexes, fully solvated pMD simulations were performed in triplicate at 25 °C (300 K) to critically compare and evaluate the molecular interactions with DNA between the A3s.

### Substrate specificity and conformation correlate with overall dynamics in the simulations

**Figure 3:**
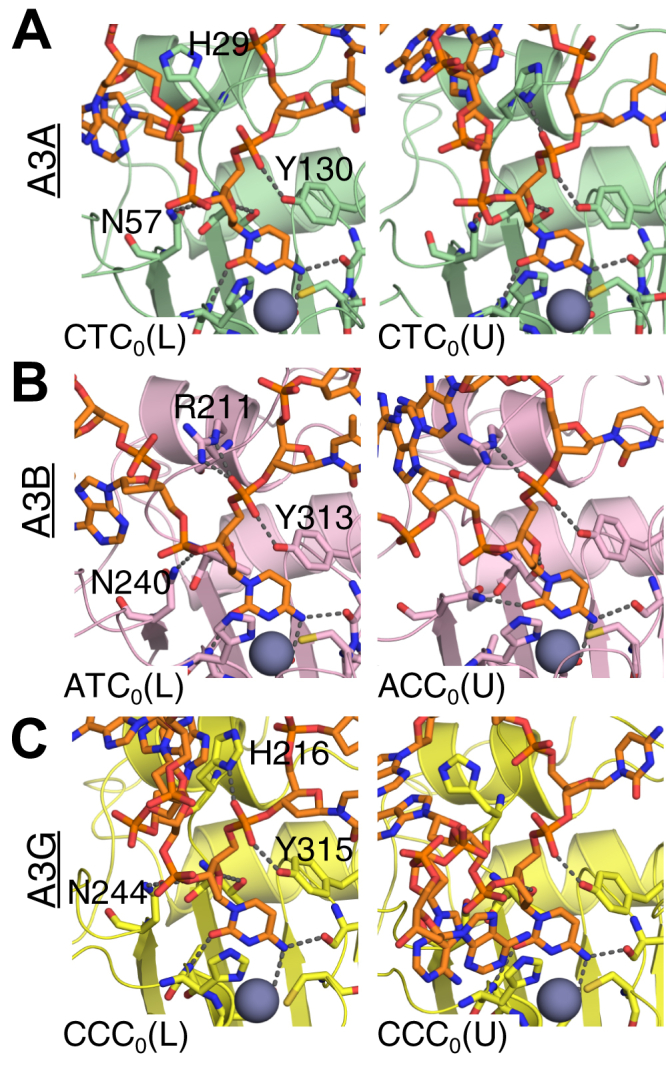
**The molecular interactions between the target cytidine (C**_**0**_**) and A3 active sites elucidate stable ssDNA bound complexes.** The final snapshot of pMD simulations where the gatekeeper residue (H29, R211, H216 for A3A, A3B and A3G, respectively) and Asn side chain coordinating the DNA are shown in *stick* representation and labeled. *A*, A3A (*green*; unstable: CTC_0_ (L), stable: CTC_0_ (U)) (*B*) A3B-CTD (*pink*; stable: ATC_0_ (L), unstable ACC_0_ (U)) (*C*) A3G-CTD (*yellow*; stable: CCC_0_ (L), unstable: CCC_0_ (U)). The A3 proteins are shown in *cartoon* representation. The active site residues that have molecular interactions with target cytidine are shown as *sticks* and labeled. ssDNA is shown as *orange sticks*. The catalytic zinc is shown as *gray sphere*.

The wild-type A3A–CTC_0_ (U) complex, which has DNA conformation in the cocrystal structure of A3A–DNA (47) with the preferred substrate sequence, was stable during the MD simulations as expected. The bound DNA had relatively small root-mean-square fluctuations (RMSF), less than 1.8 Å for the central five nucleotides (Fig. 2*A*; Fig. S2*A*). In addition, the key molecular interactions between the target cytidine (C_0_) and A3A observed in crystal structures were all well maintained throughout the course of the simulation. These included hydrogen bond interactions with His29 (gatekeeper), Thr31, Asn57, Ala71, Glu72, Ser99, Tyr130 and stacking interactions with Tyr130 and His70 (Fig. 3*A*; Fig. S3). To probe whether A3A can also stably bind ssDNA in a conformation similar to that observed in the A3G-CTD DNA-bound crystal structure, the identical substrate sequence was simulated in a linear conformation in the A3A-CTC_0_ (L) complex. The ssDNA in this complex had significantly larger fluctuations compared with A3A-CTC_0_ (U) complex (Fig. 2*A*; Fig. S2*A*), with the notable exception of the C_0_ position (RMSF A3A-CTC_0_ (U)/A3A-CTC_0_ (L): 2.9/1.6 Å at −3′, 1.6/1.1 Å at −2′, 1.1/0.7 Å at −1′, 0.5/0.6 Å at 0′, 1.8/0.9 Å at +1′ and 2.8/1.8 Å at +2′ position). In addition, the gatekeeper residue, His29, which is critical for stabilizing ssDNA binding in the U-shape seen in the crystal structure (47), flipped to the apo conformation (Fig. 3*A*; Fig. S3). As a result, the stacking interactions with downstream nucleotides (+1, +2, +3) and hydrogen bonds with the DNA backbone were lost. Together these results suggested that A3A prefers binding ssDNA bearing a CTC_0_ motif in U-shape rather than a linear conformation.

Next, A3A bound to DNA with a CCC_0_ motif, which is still deaminated but reportedly with an ∼3-fold lower efficiency compared with CTC_0_ (6), was investigated. In contrast to A3A-CTC_0_ (L), the A3A-CCC_0_ (L) complex was highly stable, comparable to the A3A-CTC_0_ (U) complex as indicated by both the RMSF of the ssDNA and stability of critical interactions with A3A (Figs. 2*A* and 3A; Figs. S2*A* and S4). The A3A-CCC_0_ (L) complex was also significantly more stable than A3A-CCC_0_ (U) as shown by the relative RMSFs (Fig. 2*A*). In fact, in the destabilized A3A-CCC_0_ (U) simulation, His70, which coordinates the catalytic zinc, lost stacking interactions with the target cytidine (70% occupancy in CCC_0_ (L); 50% occupancy in CTC_0_ (U)), appearing to destabilize the active site. Thus, A3A was able to stably bind ssDNA with both CTC_0_ and CCC_0_ motifs (6), albeit using varied interactions and with the DNA in distinct conformations: CTC_0_ motif was stable in a U-shape while the CCC_0_ motif was stable in an L-shape.

Similar to the case with A3A, the A3B-ATC_0_ (U) complex (61), which corresponds to our previously presented wild-type complex (derived from the crystal structure of chimeric A3B/A3A–DNA complex (49)), was stable during the MD simulations. However, unlike A3A, which preferred binding ssDNA depending on the target dinucleotide motif in either U- or L-shaped, A3B showed strong preference for ATC_0_ over ACC_0_ motif in MD simulations regardless of the DNA conformation. The RMSF of bound DNA in both A3B-ATC_0_ complexes, especially for −1′ and 0′ nucleotides (less than 1 Å and 0.6 Å, respectively), was relatively small compared with those in A3B-ACC_0_ complexes (Fig. 2*B*; Fig. S2). The molecular interactions between the deamination target nucleotide C_0_ and A3B were maintained only in ATC_0_ but not ACC_0_ complexes (Fig. 3*B*; Fig. S4). In the A3B-ACC_0_ (U) complex, the side chain of active site residue Asn240 changed conformation, and hydrogen bond with the ssDNA backbone was lost. This conformer change is similar to what was previously observed for A3G nonsubstrate (rC) simulations (7), which suggests that cytidine was unstable and not poised for deamination. In conclusion, A3B can accommodate both DNA conformations but strongly prefers ATC_0_ substrate.

The A3G–DNA complexes were considerably more dynamic compared with A3A and A3B, correlating with lower binding affinity (Table S2). ssDNA-binding affinity has been shown to be a good indicator of A3 activity and substrate specificity (6). The wild-type A3G–CCC_0_ (L) complex, which represents the crystal structure, was the most stable A3G complex during the MD simulations as indicated by relatively low fluctuations of bound DNA (especially the central five nucleotides) and the most stable interactions between 0′ and −1′ cytidines and protein (Fig. 2*C*; Fig. S5). In contrast, the A3G-CCC_0_ (U) complex had the highest RMSF of substrate DNA with 4.3 Å. The hydrogen bond interactions as well as the stacking interactions between His216 and ssDNA were lost in both the A3G–CTC_0_ (U) and A3G–CCC_0_ (U) complexes (Fig. 3*C*; Fig. S5). Thus, for A3G only DNA with the CCC_0_ motif in a linear conformation formed a stable complex in the simulations.

Overall, out of the 12 complexes simulated and analyzed (each of the three A3s bound to two substrate motifs in two conformations), only five A3–DNA complexes were found to be stable and further analyzed; these include A3A-CTC_0_ (U), A3A-CCC_0_ (L), A3B-ATC_0_ (U), A3B-ATC_0_ (L), and A3G-CCC_0_ (L).

### APOBEC3 preferred ssDNA-binding conformation is defined through interactions with “loop 1”

**Figure 4:**
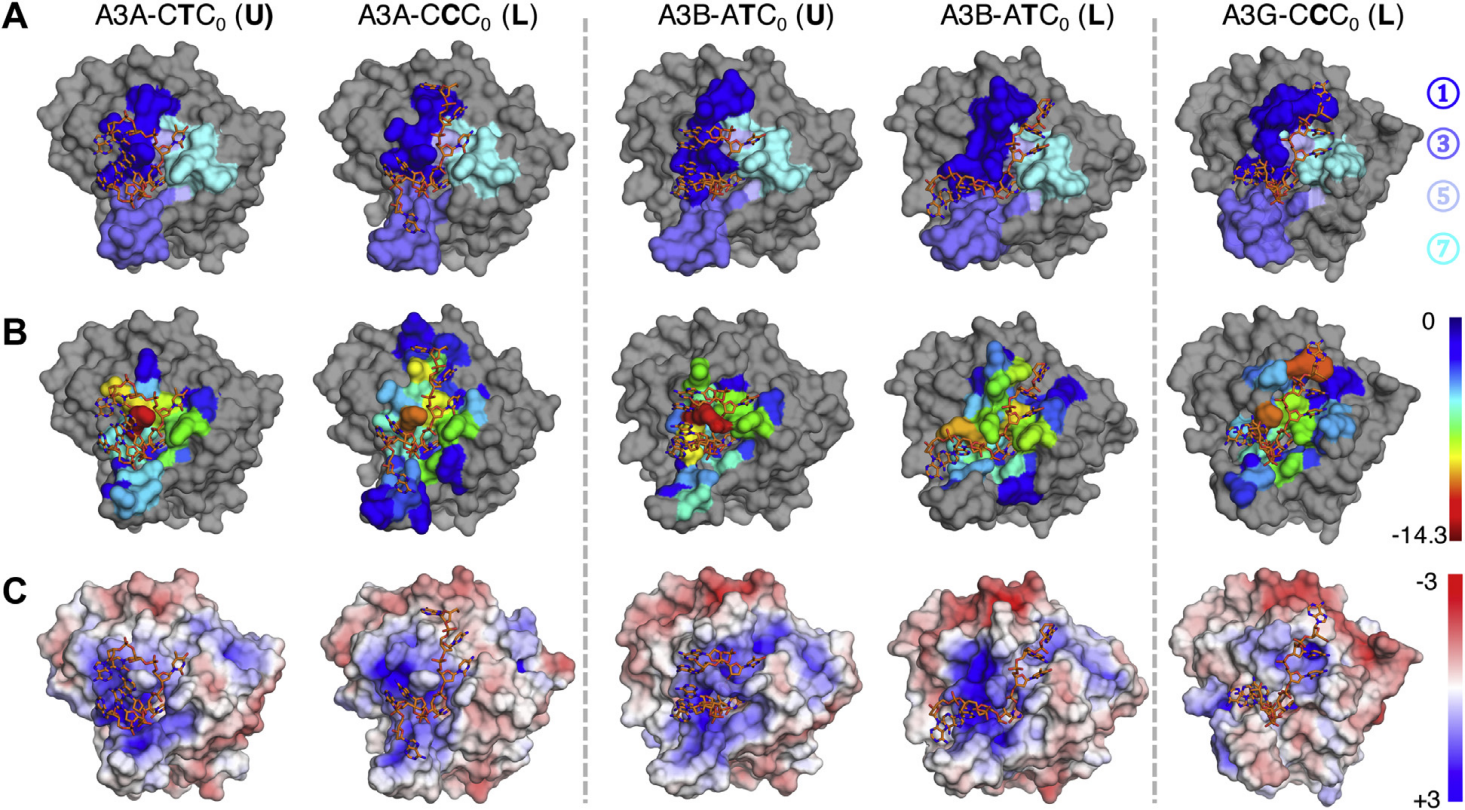
**The surface representations of the five stable ssDNA-A3 complexes reveal the importance of active site loops in substrate binding.** Active site loops and electrostatics of stable ssDNA–A3 complexes displayed for representative frames from pMD simulations. Proteins are shown in surface representation, and ssDNA is shown as *orange sticks*. *A*, active site loops (1, 3, 5, 7) are colored with different shades of *blue* and shown on protein surface. The color caption of each loop is shown on the *right*. *B*, vdW contacts between protein and ssDNA over MD simulations. The residues are colored on a rainbow scale from *blue* to *red* for increasing contacts; hence warmer colors indicate residues with the most contribution to the intermolecular contacts. The cutoff for the scale is −0.5 kcal/mol. The range of the contacts is shown on the *right*. *C*, the electrostatics of protein surfaces, where *red* is negative and *blue* is positive charge. The contour levels from positive (+3) to negative (−3).

Among all the active site loops, loop 1, whose sequence is highly variable, had the most extensive molecular contacts with ssDNA (Fig. 1). ssDNA either wrapped around (as observed in the U-shaped complexes) or extended along (as observed in the L-shaped complexes) loop 1 (Fig. 4*A*). As a result, loop 1 contributed considerable van der Waals (vdW) interactions with ssDNA to stabilize binding (Fig. 4*B*; Fig. S6). In A3A, the shorter loop 1, with a three-residue deletion relative to A3B and A3G, may allow ssDNA to bind in both conformations. In the A3A–CTC_0_ (U) complex, ssDNA wrapped around the gatekeeper residue His29 in loop 1. The neighboring Arg28 stacked with upstream bases (−2; −3) and thus stabilized the U conformation. These two residues in loop 1 had the most vdW contacts with ssDNA. In A3A–CCC_0_ (L) complex, the three-residue deletion in loop 1 allowed ssDNA to extend and make interactions with His182 in alpha-helix 6. In this binding conformation, Ile26 of loop 1 packed with upstream bases and thus had the second highest vdW interactions with ssDNA. Thus loop 1 of A3A both enabled accommodating two different DNA conformations, which permits differential specificity, and established critical interactions with the substrate in both cases.

In A3B and A3G, loop 1 is longer compared with A3A (Fig. 1*B*). In the A3G–DNA cocrystal structure, Trp211 in the 210-PWV-212 insertion stacks with the −3′ base and thus stretches the bound ssDNA into the more extended L-shape. This interaction was maintained in the complex during simulations and Trp211 was the residue with the strongest vdW interactions with DNA, indicating that Trp211 is critical for the L binding conformation of ssDNA. Similarly, in the A3B–ATC_0_ (L) complex, Pro206 of the 206-PLV-208 insertion in loop 1 packed with the −3′ nucleotide, thus stabilizing the overall extended L-shape. In A3B, binding DNA in a U-shape may be enabled by Arg210 in loop 1. Arg210 in A3B has a unique conformation compared with other A3s (40, 61): Arg210 replaces the position of the conserved Arg313 position in loop 7 and stabilizes a conserved network of hydrogen bonds initially revealed in apo A3 crystal structures as providing structural stability. As a result, the side chain of Arg210 is oriented toward the core of the protein and thus results in a cavity next to the gatekeeper residue Arg211 (61). This cavity appears to allow ssDNA to wrap around Arg211 and bind in a U conformation in the A3B–ATC_0_ (U) complex (Fig. 4*B*). Loops surrounding the active site, including loop 1, also contribute to the electrostatics of the enzyme surface to accommodate the negatively charged DNA backbone. Although still positively charged, the active site of A3G-CTD is weaker and not as positively charged compared with A3A and A3B-CTD (Fig. 4*C*), correlating with the lower binding affinity (Table S2).

### Substrate conformation and nucleotide specificity at −1′ position of the substrate DNA

**Figure 5:**
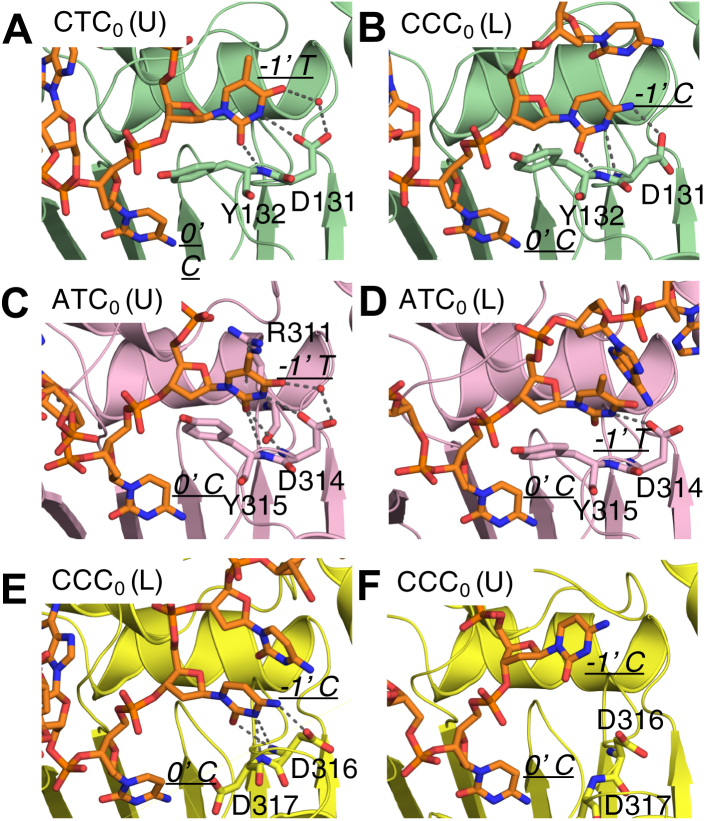
**The structural mechanism for substrate specificity at −1′ position in A3s.** A representative frame from pMD simulations for (*A* and *B*) A3A (*green*), (*C* and *D*) A3B-CTD (*pink*), (*E* and *F*) A3G-CTD (*yellow*) are used to show the molecular interactions between −1′ nucleotide and the active site. The A3 proteins are shown in *cartoon* representation. The active site residues that have molecular interactions with −1′ nucleotide base are shown as *sticks*. ssDNA is shown as *orange sticks*. The interacting residues as well as −1′ and 0 nucleotides are labeled.

We previously reported that A3A can bind either thymidine or cytidine at −1′ position with slight preference of T over C (experimental K_d_ ∼85 nM *versus* ∼250 nM) (6). Our MD simulations indicate that the TC_0_ and CC_0_ motifs are accommodated in distinct DNA-binding conformations and provide insights into preference for the TC_0_ motif: thymidine in A3A–CTC_0_ (U) complex forms stable hydrogen bonds with the side chain of Asp131 and the backbone of Tyr132 (Fig. 5*A* left and Table S3). The same hydrogen bonds are also observed in A3A–DNA cocrystal structure (47, 49). However, the C_−1_ in the unstable A3A–CCC_0_ (U) complex (see above) lost the hydrogen bond interaction with the backbone of Tyr132 and was more dynamic with RMSF about twofold higher than that of −1′ thymidine in A3A–CTC_0_ (U) (Fig. 2*A*; Fig. S2). In contrast, C_−1_ in A3A–CCC_0_ (L) complex revealed stable interactions with A3A protein (Fig. 2*A*; Fig. S2). The C_−1_ formed stable hydrogen bonds with the backbone of Tyr132 and side chain of Asp131 through a different side chain conformation (Fig. 5*A* right). The RMSF of C_−1_ in CCC_0_ (L) was about the same as T_−1_ in CTC_0_ (U) complex (Fig. 2*A*). Together these molecular interactions reveal how A3A may accommodate and deaminate TC and CC substrate sequence motifs with varied DNA-binding conformations.

**Figure 6:**
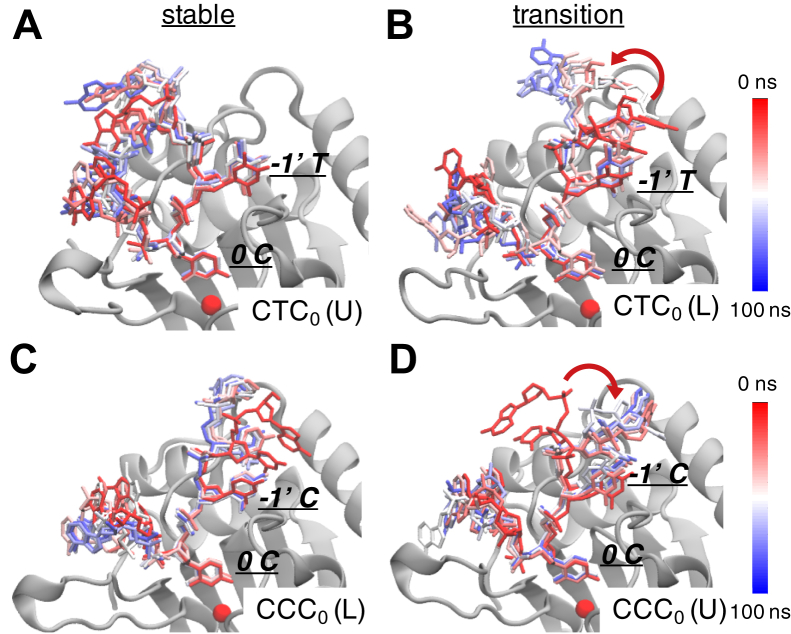
**The interdependency between substrate-binding conformation and sequence specificity at the −1′ position.** The conformational changes of ssDNA over time in A3A 37 °C MDs: (*A*) A3A-CTC_0_ (U) (*B*) A3A-CCC_0_ (U) (*C*) A3A-CTC_0_ (L) (*D*) A3A-CCC_0_ (L). In each panel, seven snapshots of the ssDNA conformation evenly spaced throughout the 100 ns MD trajectory are shown. The ssDNA is shown as *sticks* and colored by simulation time (*red* represents the beginning of the simulations while *blue* represents the end). The *red arrow* indicates the conformational transition of ssDNA to a stable position.

To further examine the interdependency between substrate-binding conformation and sequence specificity at the −1′ position, additional MD simulations with the four ssDNA-bound A3A complexes were carried out at a higher temperature of 37 °C (310.2 K). These simulations accentuated the instability of the unfavored DNA conformations and facilitated the potential for a conformational switch to a more favorable conformation. ssDNA in both A3A–CTC_0_ (U) and A3A–CCC_0_ (L) complexes was again stable during the higher temperature simulations (Fig. 6, *A* and *C*). In contrast, ssDNA initiated in A3A–CTC_0_ (L) and A3A–CCC_0_ (U) complexes underwent conformational changes to the preferred mode of binding (Fig. 6, *B* and *D*). ssDNA in the A3A–CTC_0_ (L) complex transitioned from an L-shape to U-shape, while ssDNA initiated in a A3A–CCC_0_ (U) conformation transitioned from U-shape to L-shape during the simulations. These additional simulations not only validated our results at 25 °C but also supported the interdependency between ssDNA-binding conformation and substrate sequence specificity for A3A.

A3B exhibits stronger preference for T rather than C at the −1′ position, unlike A3A (53). However, for A3A and A3G, the complexes analyzed above had a C at the upstream −2′ position. To investigate any influence of −2′ nucleotide on substrate preference, additional A3B complexes with CTC_0_ motif were characterized and compared with ATC_0_ motif. When the DNA was in a U-shape, *i.e.*, in both A3B–ATC_0_ (U) and A3B-CTC_0_ (U) complexes, −1′ T formed stable hydrogen bonds with the backbone of Tyr315 and the side chain of Asp314, which was similar to what was observed in A3A and consistent with our previous results (61) (Fig. 5*B* left; Table S3). In the A3B–ATC_0_ (L) complex, unlike what was observed in A3A–CTC_0_ (L), Asp314 maintained the same side hydrogen bond as in U-shape conformation and thus promoted T over C (Fig. 5*B* right; Table S2). Thus, these results indicate that within an ATC_0_ motif A3B can accommodate T at −1′ position in either DNA conformation.

A3G binds ssDNA in an L shape and thus prefers CC_0_ rather than TC_0_ at −1′ position. A3G is the only A3 that prefers cytidine over thymidine at −1′ position. In our MD simulations of the A3G-CCC_0_ (L) complex, the side chain of Asp316 and the backbone of Asp317 formed stable hydrogen bonds with −1′ cytidine (Fig. 5*C* left; Table S3). These direct hydrogen bonds were lost in the unstable A3G–CCC_0_ (U) complex simulation (Fig. 5*C* right; Table S3), further confirming the preference of A3G-CCC_0_ for the more stable L-shaped conformation.

### Nucleotide specificity at −2′ position of substrate DNA

**Figure 7:**
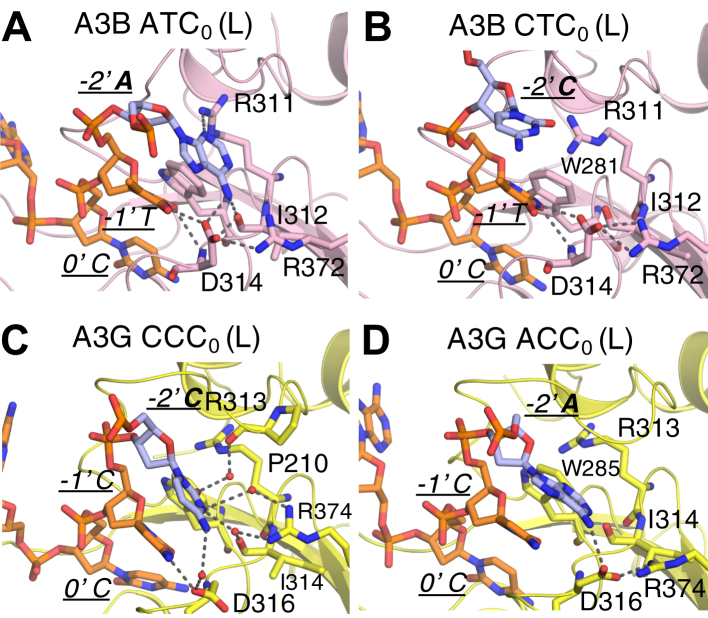
**The structural mechanism for substrate specificity at −2′ position in A3s.** A representative frame from MD simulations for (*A*) A3B-CTD (*pink*), (*B*) A3G-CTD (*yellow*) are used to show the molecular interactions between −2′ nucleotide and A3s. The residues that have molecular interactions with −2′ nucleotide base are shown as *sticks*. ssDNA is shown as *orange sticks* except −2′ nucleotide, which is colored in *blue*. −2′, −1′ and 0′ positions as well as the residues contacting −2′ nucleotide are labeled.

Intra-DNA interactions in A3A, as we previously revealed, may underlie the substrate specificity of T/C at −2′ position (6). However, atomic interactions defining the −2′ specificity for A3B or A3G have remained elusive. To address the molecular mechanism of specificity at −2′ position in A3B, additional models of A3B with a C at position −2′ (A3B–CTC_0_) and (A3B–CCC_0_) were compared with A3B’s preferred ATC sequence (53) which was used in the complexes described above. In A3B–ATC_0_ (L) complex simulation, A_−2_ formed stable hydrogen bonds with the side chain of Arg311 (occupancy 38% during the simulations), backbone of Ile312 (occupancy 90%), Asp314 (occupancy 98%), and stacking interactions with Trp281 (occupancy 32%) (Fig. 7*A*). However, all these interactions were significantly weaker in the A3B–CTC_0_ (L) complex simulations (Fig. 7*B*) especially for Trp281. Moreover, the side chain of the gatekeeper residue for DNA binding, Arg211, lost interactions with DNA backbone, which likely impairs the binding of target cytidine C_0_ in the active site (Fig. S4). The destabilization of these interactions is consistent with preference of A3B for the ATC over CTC sequence.

To investigate the specificity at −2′ position for A3G, a model with ACC trinucleotide motif was compared with CCC, which is the preferred motif for A3G (30, 66, 67, 68). In the A3G–CCC_0_ (L) complex simulation, C_−2_ was locked in an extensive hydrogen bonding network with residues Pro210 (water-mediated; occupancy 37%), Arg374 (water-mediated; occupancy 31%, 41%), backbones of Ile314 (water-mediated; occupancy 41%), and Val212 (water-mediated; occupancy 34%) and Asp316 (occupancy 43%) (Fig. 7*C*). Even though there is enough space to accommodate an A, the larger base of A in the ACC_0_ (L) complex simulation did not occupy the space where water-mediated hydrogen bonds had been; instead, the A_−2_ was significantly destabilized and formed only one hydrogen bond with Asp316 (occupancy 42%) (Fig. 7*D*). Thus, A3G preferred to accommodate the smaller C at the −2′ position through an extensive water-mediated hydrogen bonding network.

## Discussion

In this study, we investigated the structural molecular mechanism for substrate specificity in A3 enzymes and found an interdependence between substrate conformation and specificity. Specifically, our results indicate that A3A and A3B can accommodate DNA in a linear conformation, in addition to the wrapped U-shaped binding conformation of substrate DNA observed in crystal structures. For A3A, the linear conformation permits recognition of CC_0_ dinucleotide motif while the U-shape prefers TC_0_ as observed in the crystal structure. The active site loops are key in defining the overall binding surface and conformation adopted by the DNA to bind A3s. For A3A, A3B, and A3G, loop 1 is critical with extensive interactions with DNA including the gatekeeper residues (His29 in A3A, Arg211 in A3B, and His216 in A3G), which locks DNA in the active site. We described that the three-residue insertion in loop 1 of A3B and A3G compared with A3A underlies the preference for the more extended L-shaped conformation of the DNA through specific stacking interactions. These results indicate that DNA conformation should be considered together with nucleotide preference in defining substrate specificity of A3 enzymes.

Another key loop in substrate recognition is loop 7; previous studies have shown that swapping loop 7 from A3G into A3B altered the substrate specificity in A3B from TC_0_ to CC_0_ (40). Additionally, changing Asp317 of A3G into the corresponding residue of A3A (Tyr132) caused A3G to adopt a more A3A-like 5’-TC_0_ preference (69). While these results suggested that Tyr132 (Tyr315 in A3B) in loop 7 might be important for substrate specificity at −1’ position, our analysis demonstrates there is no specific hydrogen bond between this residue and −1′ base during the MD simulations. Instead we propose this tyrosine is key for stabilizing the U-shaped binding conformation of DNA, and thus the TC_0_ preference in A3A/A3B and not in A3G. This is supported by the significantly higher vdW contacts of Tyr132/315 with DNA compared with those of Asp317 in A3G (Fig. S6). Thus tyrosine at this critical position in loop 7 stabilizes the U-shape of bound DNA, allowing accommodation of a T at this position.

Unlike human CDAs that bind single nucleosides (70), A3s require at least five consecutive nucleotides for stable substrate recognition (6, 30, 71). The interdependence between binding interactions around the active site suggests that the sequence of nucleotides flanking the target cytidine is also critical for stable substrate binding. We observed interdependence between preference and interactions of various nucleotides positions in our pMD analysis. The nucleotide at −1′ position affects the binding stability of the target nucleotide in the catalytic site (0 position). Having a disfavored nucleotide at the −1′ position destabilized the target nucleotide. Additionally, the nucleotide at −2′ position can influence the specificity at −1′ position. In A3B, the interactions between −2′ A and Asp314 locked the side chain in a conformation that promotes thymidine over cytidine at −1′ position. In both A3A and A3B, preference for T at the −1 position was possible only when the substrate DNA was in a U-shape, which cannot be accommodated by A3G.

Considering the roles of A3s in viral infections and cancer, a better understanding of the molecular mechanism by which A3s recognize different oligonucleotides will be critical for developing therapeutics. Currently, combined with catalytically inactive Cas9 (dCas9), A3s are investigated as novel base editors for direct modification of genomic DNA at single-base resolution (21, 22); as cytosine base editors (CBE), A3s can create mutations to potentially correct genetic diseases. Nevertheless, these editors still require optimization, considering that CBEs can have significant off-target effects, low purity, and wide editing windows (72). To overcome these problems, several versions of CBEs have been engineered; UGI added to increase product purity (73) and generate high fidelity Cas9 with reduced off-target effects (74) or different Cas nucleases (75, 76, 77, 78, 79) were used to narrow activity window. However, modifications to improve the efficiency and fidelity of base editing have not focused on the deaminase. Another major problem in applying CBEs to treat genetic diseases is that the target site must naturally exist in the preferred DNA sequence context for the cytidine deaminase, which may or may not be the case for the desired modification. Therefore, having a library of A3s with different substrate specificities as context-dependent base editors would expand the toolkit available for targeted base editing. Our results revealing the molecular mechanisms underlying A3 specificities could help guide engineering of A3s, especially with modifications to the active site loops, to rationally design A3s with the desired sequence specificity.

## Experimental procedures

### Protein sequence alignment

Protein sequence alignment was generated by program Geneious 9.0.5 using default multiple alignment.

### Molecular modeling

All structure models in this study were first generated with MODELLER9.23 and then optimized using Protein Preparation Wizard in Maestro from Schrodinger suite. The optimization was performed at pH 7.0 and minimized using restrained minimization panel. The ssDNA-bound crystal structures of A3A (PDB: 5KEG for protein; 5SWW for ssDNA) and A3G-CTD2 (PDB: 6BUX) were used as templates for molecular modeling of wild-type A3-ssDNA complexes. ssDNA sequences were mutated through program Coot. ssDNA-bound A3B-CTD structures were modeled using the crystal structures of both apo A3B-CTD (PDB: 5CQH) and A3A-ssDNA complex.

### Molecular dynamics simulations

All molecular dynamics simulations were performed for 100 ns using program Desmond from the Schrodinger suite. The simulation systems were built using SPC solvation model and cubic boundary conditions of 12 Å buffer box size with OPLS3 force field through system builder in Maestro. The final systems were neutral and had 0.15 M sodium chloride. A multistage MD simulation protocol was used, which was previously described (61), at 298 °K (25 °C). The four A3A-ssDNA models were also simulated at 310.2 °K (37 °C) using the same simulation protocol.

### Analysis of molecular dynamics simulations

The RMSD and molecular interactions (hydrogen bond, stacking interaction occupancies) over the trajectories were calculated using Simulation Interaction Diagram in Maestro from Schrodinger. The per base RMSFs of ssDNA were calculated using Schrodinger python API. The residue vdW potential between A3s and ssDNA during the MD simulations was extracted from the simulation energies using Desmond.

The frame closest to the average RMSD was used as a representative structure for each MD simulation. The electrostatic distributions were calculated using PDB2PQR server and Pymol with the APBS plugin and visualized with contour levels positive (+3) and negative (−3). The time series representations of ssDNA were generated with program VMD using 2000 frames as time step (total 20,000 frames for each MD trajectory). All other structural graphics were made using the program PyMol.

## Supporting information

This article contains supporting information.

## Conflict of interest

The authors declare no conflict of interest.
