# Supplementary material for: Structural basis of substrate specificity in human cytidine deaminase family APOBEC3s
Source: J Biol Chem. 2021 Jun 24;297(2):100909. doi: 10.1016/j.jbc.2021.100909 (PMC8313598; doi:10.1016/j.jbc.2021.100909)
Supplement: Supplemental Figures S1–S6 and Tables S1–S3 [file mmc1.docx]

**Supplementary Information**

**Structural mechanism of substrate specificity in human cytidine deaminase family APOBEC3s**

Shurong Hou^1^, Jeong Min Lee^1^, Wazo Myint^2^, Hiroshi Matsuo^2^, Nese Kurt Yilmaz^1*^, Celia A. Schiffer^1*^

^1^Department of Biochemistry and Molecular Pharmacology, University of Massachusetts Medical School, Worcester, MA 01655, USA

^2^Basic Research Laboratory, Frederick National Laboratory for Cancer Research, Frederick, MD 21702, USA

*Joint corresponding author: Celia A. Schiffer, Nese Kurt Yilmaz

E-mail: [Celia.Schiffer@umassmed.edu](mailto:Celia.Schiffer@umassmed.edu); Nese.KurtYilmaz@umassmed.edu

**Running title**: Substrate Specificity of APOBEC3s

**Keywords**: APOBEC3, specificity, structural analysis, molecular modeling, molecular dynamics simulation

**Supplementary Table 1.** List of A3-DNA complexes for which MD simulations and analysis were performed in this study. All three A3 enzymes had full wild-type (WT) sequence, with any substitution in the crystal structure construct reverted back to the native amino acid. In ssDNA sequence, the underlined C denotes the target C (C_0_ in abbreviation) while U and L indicate U-shaped and L- shaped ssDNA conformation, respectively.

| **Protein** | **ssDNA** | **Abbreviation** |
| --- | --- | --- |
| A3A WT | AC**TC**AAA (U) | A3A-CTC_0_ (U) |
|  | AC**TC**AAA (L) | A3A-CTC_0_ (L) |
|  | AC**CC**AAA (U) | A3A-CCC_0_ (U) |
|  | AC**CC**AAA (L) | A3A-CCC_0_ (L) |
| A3B-CTD WT | A**ATC**AAA (U) | A3B-ATC_0_ (U) |
|  | A**ATC**AAA (L) | A3B-ATC_0_ (L) |
|  | A**ACC**AAA (U) | A3B-ACC_0_ (U) |
|  | A**ACC**AAA (L) | A3B-ACC_0_ (L) |
|  | A**CTC**AAA (U) | A3B-CTC_0_ (U) |
|  | A**CTC**AAA (L) | A3B-CTC_0_ (L) |
|  | A**CCC**AAA (U) | A3B-CCC_0_ (U) |
|  | A**CCC**AAA (L) | A3B-CCC_0_ (L) |
| A3G-CTD WT | AC**TC**AAA (U) | A3G-CTC_0_ (U) |
|  | AC**TC**AAA (L) | A3G-CTC_0_ (L) |
|  | AC**CC**AAA (U) | A3G-CCC_0_ (U) |
|  | AC**CC**AAA (L) | A3G-CCC_0_ (L) |
|  | A**ACC**AAA (L) | A3G-ACC_0_ (L) |

**Supplementary Table 2.** Binding affinity (equilibrium dissociation constant K_d_) for linear and hairpin ssDNA with preferred nucleotide sequence by A3s.

| **Protein** | **ssDNA** | **K_d_** |
| --- | --- | --- |
| A3A | AAA-ACC_0_-AAA-AAA (Linear) ^1^ | 250 ±14 nM |
|  | AAA-ATC_0_-AAA-AAA (Linear) ^1^ | 145 ± 2 nM |
|  | AAA-ATC_0_-GAA-AAA (Linear) ^1^ | 154 ± 2 nM |
|  | AAA-CTC_0_-AAA-AAA (Linear) ^1^ | 85 ± 1 nM |
|  | G-CCA-TCA-TTC_0_-GATG-GG (hairpin) ^1^ | 26 ± 2 nM |
| A3B-CTD | A-AAA-AAA-ATC_0_-GAA-AA (Linear) ^2^ | 5.4 ± 2.6 µM |
|  | G-CCA-TCA-TTC_0-_GATG-GG (hairpin) ^2^ | 2.0 ± 0.5 µM |
| A3G-CTD | AAT-CCC_0_-AAA (Linear) ^3^ | 160 µM |

^1^ Silvas et al., *Scientific Reports* 8.1 (2018): 7511.

^2^ Hou et al., *JCTC* 15.1 (2018): 637-647.

^3^ Maiti et al., *Nat Commun* 9.1 (2018): 2460.

**Supplementary Table 3.** Hydrogen bond frequencies form MD simulations between active site residues of A3 and -1’ nucleotide of ssDNA. Water-mediated hydrogen bonds are shown with underlines. Lost/absent interactions are indicated in gray.

| **System (U)** | **Residue** | **Occupancy** | **System (L)** | **Residue** | **Occupancy** |
| --- | --- | --- | --- | --- | --- |
| A3A-CTC_0_ (U) | Asp131 | 99%; 76% | A3A-CTC_0_ (L) | Asp131 | 79% |
|  | Tyr132 | 84% |  | Tyr132 |  |
| A3A-CCC_0_ (U) | Asp131 | 85%; 34% | A3A-CCC_0_ (L) | Asp131 | 82%; 42% |
|  | Tyr132 |  |  | Tyr132 | 96% |
| A3B-ATC_0_ (U) | Asp314 | 99%; 42%; 57% | A3B-ATC_0_ (L) | Asp314 | 100% |
|  | Tyr315 | 45% |  | Tyr315 |  |
|  | Arg311 | 57%; 61% |  | Arg311 |  |
| A3B-ACC_0_ (U) | Asp314 | 57% | A3B-ACC_0_ (L) | Asp314 | 89% |
|  | Arg311 | 70%; 55% |  | Arg311 |  |
| A3G-CTC_0_ (U) | Asp316 | 81%; 70%; 72% | A3G-CTC_0_ (L) | Asp316 | 71%; 32%; 35% |
|  | Arg374 | 55% |  | Asp317 | 80% |
|  | Pro210 | 43% |  |  |  |
| A3G-CCC_0_ (U) | Asp316 | 60% | A3G-CCC_0_ (L) | Asp316 | 54%; 36% |
|  | Asp317 | 35% |  | Asp317 | 58% |


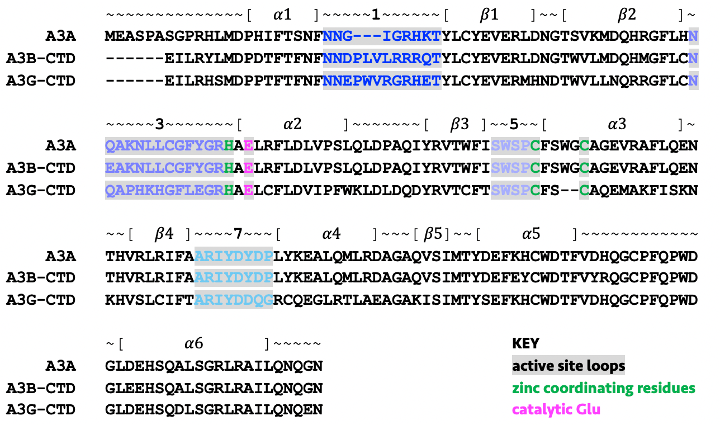


**Supplementary Figure 1.** Amino acid sequence alignments of wild type A3A, A3B-CTD and A3G-CTD. Secondary structure elements (alpha helices α and beta strands β) are indicated on top of the sequences. Active site loops are colored in the same shade of blue as in Figures 1 and 4.

**Supplementary Figure 2.** Snapshots of ssDNA conformation from MD simulations. All twelve simulations with 3 enzymes, 2 target motifs, and 2 ssDNA conformations are shown. Figure 2 of the main manuscript includes 6 selected out of these 12 simulations. In each panel, 7 snapshots of the ssDNA conformation evenly spaced throughout the 100 ns MD trajectory are shown. The A3 proteins are in grey cartoon representation. The ssDNA is shown as stick; colored based on the simulation time from red to blue. The target (0 C) and -1’ position nucleotide in the active site are labeled. The catalytic zinc is shown as red sphere.

**Supplementary Figure 3.** The comparison of the first and final frame from A3A simulations. Snapshots of ssDNA (orange sticks) and protein (green cartoon) at the start and end of the MD simulations are shown in the first two rows and superimposed in the last row. Side chain of active site residues that interact with backbone are shown as sticks and labeled. Hydrogen bonds are indicated by dashed lines, and the catalytic zinc is shown as gray sphere.

**Supplementary Figure 4.** The comparison of the first and final frame from A3B simulations. Snapshots of ssDNA (orange sticks) and protein (pink cartoon) at the start and end of the MD simulations are shown in the first two rows and superimposed in the last row. Side chain of active site residues that interact with backbone are shown as sticks and labeled. Hydrogen bonds are indicated by dashed lines, and the catalytic zinc is shown as gray sphere.

**Supplementary Figure 5.** The comparison of the first and final frame from A3G simulations. Snapshots of ssDNA (orange sticks) and protein (yellow cartoon) at the start and end of the MD simulations are shown in the first two rows and superimposed in the last row. Side chain of active site residues that interact with backbone are shown as sticks and labeled. Hydrogen bonds are indicated by dashed lines, and the catalytic zinc is shown as gray sphere.

**Supplementary Figure 6.** Interaction of A3 active site residues with ssDNA in pMD simulations quantified by vdW energies for (**A**) A3A (**B**) A3B-CTD (**C**) A3G-CTD. Color coding according to the target dinucleotide motif and ssDNA conformation (U or L shape) is given at the top of the figure. Residues corresponding to the active site loop 1 are indicated by the orange brackets.
